# Coaxial Synthesis of PEI-Based Nanocarriers of Encapsulated RNA-Therapeutics to Specifically Target Muscle Cells

**DOI:** 10.3390/biom12081012

**Published:** 2022-07-22

**Authors:** Raquel de la Hoz, Nazely Diban, María T. Berciano, Carlos San Emeterio, Ane Urtiaga, Miguel Lafarga, José C. Rodríguez-Rey, Olga Tapia

**Affiliations:** 1 Department of Chemical and Biomolecular Engineering, University of Cantabria, 39011 Santander, Spain; raquel.delahoz@unican.es (R.d.l.H.); ana.urtiaga@unican.es (A.U.); 2 Health Research Institute Valdecilla (IDIVAL), 39011 Santander, Spain; berciant@unican.es (M.T.B.); lafargam@unican.es (M.L.); josecarlos.rodriguez@unican.es (J.C.R.-R.); 3 Department of Molecular Biology, University of Cantabria, 39011 Santander, Spain; 4 Centro de Investigación Biomédica en Red Sobre Enfermedades Neurodegenerativas (CIBERNED), 28029 Madrid, Spain; 5 Research Group on Food, Nutritional Biochemistry and Health, Universidad Europea del Atlántico, 39011 Santander, Spain; carlos.sanemeterio@alumnos.uneatlantico.es; 6 Department of Anatomy and Cellular Biology, University of Cantabria, 39011 Santander, Spain

**Keywords:** nanoparticle, coaxial electrospraying, polyethylenimine, RNA-therapeutics, aptamers, muscle-specific therapy, spinal muscular atrophy, Nusinersen

## Abstract

In this work, we performed a methodological comparative analysis to synthesize polyethyleneimine (PEI) nanoparticles using (i) conventional nanoprecipitation (NP), (ii) electrospraying (ES), and (iii) coaxial electrospraying (CA). The nanoparticles transported antisense oligonucleotides (ASOs), either encapsulated (CA nanocomplexes) or electrostatically bound externally (NP and ES nanocomplexes). After synthesis, the PEI/ASO nanoconjugates were functionalized with a muscle-specific RNA aptamer. Using this combinatorial formulation methodology, we obtained nanocomplexes that were further used as nanocarriers for the delivery of RNA therapeutics (ASO), specifically into muscle cells. In particular, we performed a detailed confocal microscopy-based comparative study to analyze the overall transfection efficiency, the cell-to-cell homogeneity, and the mean fluorescence intensity per cell of micron-sized domains enriched with the nanocomplexes. Furthermore, using high-magnification electron microscopy, we were able to describe, in detail, the ultrastructural basis of the cellular uptake and intracellular trafficking of nanocomplexes by the clathrin-independent endocytic pathway. Our results are a clear demonstration that coaxial electrospraying is a promising methodology for the synthesis of therapeutic nanoparticle-based carriers. Some of the principal features that the nanoparticles synthesized by coaxial electrospraying exhibit are efficient RNA-based drug encapsulation, increased nanoparticle surface availability for aptamer functionalization, a high transfection efficiency, and hyperactivation of the endocytosis and early/late endosome route as the main intracellular uptake mechanism.

## 1. Introduction

RNA-based therapies are emerging as a powerful technology for treating diseases ranging from genetic disorders to viral infections and some types of cancer. They involve the use of RNA drugs, including small interfering RNAs (siRNAs), aptamers, ribozymes, or antisense oligonucleotides (ASOs), among others, to correct gene expressions [1]. These RNA therapeutics can interfere with several gene expression regulatory mechanisms via the impairment of mRNA translation into functional deleterious proteins, the induction of RNase H-dependent mRNA degradation, the inhibition of pre-mRNA 5′-cap formation, the modulation of aberrant RNA splicing, or the blockade of polyadenylation [2,3,4].

ASOs are short, single-stranded RNA synthetic molecules (between 15 and 50 nucleotides) that bind with Watson–Crick base-pairing complementarily to both nuclear and cytoplasmic-located target RNA to influence RNA processing and modulate the protein expression [2,3]. Therapeutic ASOs must be able to travel through the circulatory system, be incorporated by the disease-targeted cells, and be released into the cytoplasm without being degraded by endoribonucleases or filtered by the kidneys [2,4]. The intrinsic negative charge of the ASOs prevents their cellular uptake through the cell membrane, which is protected by the anionic barrier of the glycocalyx [5] and could trigger an immune response [4]. To avoid the instability of using RNA molecules, researchers have developed second-generation ASOs with chemical modifications of the backbone chemistry and on the 2′ position of the ribose that further enhance its pharmacological and therapeutic properties and mRNA-binding affinity [2,6,7]. Most therapeutic ASOs usually combine phosphorothioate group (PS) linkages with modifications at the 2′ position of the ribose, e.g., 2′–O–methoxy–ethyl (2′-MOE) modification [8].

To favor target delivery and minimize in vivo ASO degradation when directly administered, high ASO concentrations are used. This could lead to the appearance of some adverse reactions [9]. Instead, the use of synthetic carriers is considered an efficient alternative to overcome the drawbacks of ASO delivery into the affected cells. Oligonucleotide carriers can be classified into viral and nonviral vectors. Although viral vectors have an efficient delivery capacity, they present problems, such as a limited gene carrier capacity, high large-scale production cost, immunogenicity, and insertional mutagenesis [10,11]. Therefore, nonviral vectors (e.g., cationic lipids, dendrimers, cationic polymers, and inorganic nanoparticles) have attracted special attention despite their low transfection efficiency [11,12].

Biodegradable cationic polymers (e.g., polyethylenimine (PEI), chitosan, poly-L-lysine, and poly(2-N-(dimethylaminoethyl)-methacrylate)) are nonviral gene delivery systems that have a tunable time of drug release and proven gene delivery efficiency [13]. Amongst them, PEI has been widely studied due to its exceptional ability to condensate DNA and RNA molecules into stable nanocomplexes via electrostatic interactions between the phosphate group of the oligonucleotides and primary amines of the PEI. These PEI/RNA nanocomplexes are formulated to be incorporated into target cells by endocytosis [14], and the inherent strong positive density of PEI mediates RNA release into the cytoplasm [15,16]. However, the cellular uptake of PEI-based nanocomplexes is sometimes limited [17,18]. The interaction of PEI with the cell membrane and, therefore, the transfection efficiency of PEI nanocomplexes has been improved by modifying the PEI surface with long, lipophilic substituents (e.g., palmitic acid or hexyl or dodecyl chains). Furthermore, some studies revealed that aptamers, RNA- or DNA-based oligonucleotides (between 20 and 50 nucleic acids in length), are promising molecules for enhancing cell specificity, as they could be designed to specifically bind cell type-specific surface molecules with minimal toxicity and null immunogenicity [17,19]. 

There are different techniques that are widely used for producing polymer-based nanocomplexes for gene therapy, such as solvent evaporation, salting-out, nanoprecipitation, or oil/water emulsion [20]. The most widespread method for synthetizing nano-sized particles is conventional nanoprecipitation, also known as the bulk mixing method. This technique is based on the dropwise addition of a polymeric solution to an aqueous phase under constant stirring. Although it is a straightforward method, the nanocomplex size is not well-controlled, and the transfection efficiency could be affected [21]. An alternative technology to produce monodisperse biopolymeric nanocomplexes with a controlled size is electrospraying [22]. Recently, coaxial electrospraying has received special attention for producing core–shell nanoparticles for drug and gene delivery in biomedical applications, including the successful encapsulation of genetic material (DNA and RNA), cells, or proteins without changing the biological activity of these materials [21,22,23]. This technique allows a one-step production and external functionalization of drug-encapsulating polymeric nanocomplexes. 

In the present study, we aimed to perform a comparative analysis of different methodologies to synthesize nonviral polymeric (PEI-cit) nanocomplexes to be used as vectors for intracellular ASO delivery. Three different synthesis methodologies were compared to prepare the PEI-based ASOs nanocomplexes (PEI-cit/ASO) externally functionalized with muscle-specific RNA aptamers (PEI-cit/ASO/Ap): (i) conventional nanoprecipitation (NP), (ii) electrospraying (ES), and (iii) coaxial electrospraying (CA). The size and uniformity of the PEI-cit, PEI-cit/ASO, and PEI-cit/ASO/AP nanocomplexes were characterized, as well as the Z-potential, by dynamic light scattering (DLS) and transmission electron microscopy (TEM). Furthermore, the transfection efficiency of the functionalized nanocomplexes was also assessed, both qualitatively and quantitatively, in C2C12 muscle cells.

## 2. Materials and Methods

### 2.1. Synthesis of PEI-Cit/ASO Nanocomplexes

Three methodologies to synthesize the PEI-cit/ASO nanocomplexes were used and compared in the present work. All procedures were performed at room temperature unless otherwise stated.

(i) Conventional nanoprecipitation method (NP): A 2.8 μM sodium citrate solution was added drop-wise to a 100 μg/mL solution of branched 60 kDa PEI (Sigma Aldrich, St. Louis, MO, USA), pH 6.0, with hydrochloric acid (0.1 M) at a 6:1 volume ratio, respectively, under magnetic stirring. The PEI-cit reaction was maintained under moderate stirring over 20 min. When PEI-cit nanocomplexes were functionalized with ASOs (nontargeting sequence 5′-TCATTTGCTTCATACACAGG-3′, IDT-Dna Tech. [24]), the mixture of PEI and sodium citrate was allowed to react only for five minutes. Afterwards, the ASO solution (6.65 μM) was added drop-wise to the freshly formed PEI-cit nanocomplexes until obtaining a molecular ratio of 3:1 (PEI:ASO). The reaction was maintained under moderate stirring for 15 min. The ASO concentration for the nanocomplex synthesis was set at 6.65 µM in order to obtain a stock solution of PEI-cit/2 µM ASO nanocomplexes. Stock nanocomplexes solutions was kept at 4 °C until used (Figure S1A).

(ii) Electrospraying (ES): A commercial ES set-up (Starter Kit-Aligned, Linari Nano-tech, Pisa, Italy) was employed. A 2.8 μM sodium citrate solution was extruded with a syringe pump throughout a metallic capillary needle with a 0.82-mm internal diameter at a controlled flow rate of 6 mL/h. A voltage of 20 kV was applied between the needle and a grounded steel plate collector separated by 3.7 cm. The sprayed droplets of the sodium citrate solution were collected in 1.8 mL of a PEI solution (100 μg/mL) that was maintained under continuous stirring. A sodium citrate:PEI volume ratio of 6:1 was used. PEI-cit nanocomplexes were magnetically stirred for five minutes, and subsequently, ASO solution (6.65 μM) was added dropwise until obtaining a molecular ratio of 3:1 (PEI:ASO) (Figure S1A). Finally, the stock solution of the PEI-cit/2 µM ASO nanocomplexes solution was kept under stirring for an additional 15 min. As the negative control, PEI-cit nanocomplexes without ASO functionalization were stirred for 20 min until there was a complete reaction. Stock nanocomplex solutions were kept at 4 °C until used (Figure S1A).

(iii) Coaxial electrospraying (CA): The operating mode employs the experimental commercial electrospraying system described above. A coaxial needle was used with a 0.83-mm inner diameter and 1.83-mm outer diameter. The ASO solution (15.2 μM) flowed through the inner needle, and the PEI solution (100 μg/mL) flowed throughout the outer needle at the same flow rate of 1 mL/h. As both flow rates had to be equal to assure a stable cone-jet mode, the concentration of the ASO solution was adjusted to 15.2 μM in order to maintain the final concentration of the stock PEI-cit/2 µM ASO nanocomplexes solution consistent throughout all the synthesis techniques. The applied voltage was 17 kV, and the needle-to-collector distance was 6 cm. The coextruded PEI and ASO solutions were sprayed into 1.8 mL of a 2.8 μM sodium citrate solution and stirred for an additional 15 min. Stock nanocomplex solutions were kept at 4 °C until used (Figure S1B).

### 2.2. Functionalization of PEI-Cit/ASO Nanocomplexes with APTAMERS

The PEI-cit/ASO nanocomplexes were externally functionalized with the Cy3-conjugated aptamer A01B (IDT-Dna with 5′-Cy3 dye, 2′-Fluorine chemically modified pyrimidines, and a phosphodiester backbone) [25]. The Cy3-conjugated A01B powder was resuspended in Tris-EDTA (TE) buffer (10 mM Tris-HCl, pH 8, and 1 mM EDTA). Prior to use, 10 μL of 100 μM A01B aptamer were mixed with 90 μL of folding buffer (1 mM MgCl_2_ and DPBS 1×, pH 7.5) to adopt its functional secondary structure. The folding conditions were as follows: 10 min at 70 °C, chilled on ice for 5 min, and slowly cooled at 37 °C for 30 min. A small amount (30 μL) of PEI-cit/ASO nanocomplex solution was mixed with 10 μL of folded Cy3-A01B aptamer solution and incubated for 15 min (Figure S1A,B).

For the preparation of fluorescent FITC-PEI-cit/ASO nanocomplexes, a solution of 10 mM FITC fluorophore diluted in EtOH was mixed with 10 mg/mL of the polymeric solution in a volume ratio of 5:1 of PEI. The FITC-PEI reaction was kept for 2.5 h at 18 °C in dark conditions. To remove the nonlinked FITC, a dialysis protocol was employed: the dialysis membrane (MWCO 3.5 kDa) was soaked in deionized water for 15–30 min prior to use. Next, the FITC-PEI mixture was charged into the dialysis membrane and was dialyzed twice against 2 L of cold deionized water for 2 h at room temperature in dark conditions and, finally, overnight. The fluorescence of all the dialysates was measured by an UV-1800 UV–Vis spectrophotometer (Shimadzu, Kyoto, Japan) at a wavelength of 490 nm. Afterwards, the nanocomplexes with FITC-PEI were synthesized by the three synthesis techniques (NP, ES, and CA) and further functionalized with Ap-Cy3 following the same methodology as the previously mentioned protocol.

### 2.3. Nanocomplexes Characterization

The particle size distribution and Z-potential of the nanocomplexes were measured by dynamic light scattering (DLS) and the electrophoresis principle using a Zetasizer Nano ZS (Malvern Panalytical, Malvern, UK) at 25 °C using a 4-mW He-Ne laser operating at 633 nm.

Transmission electron microscopy (TEM) images were also acquired to determine the subcellular localization of the nanocomplexes. The nanocomplex samples were preconcentrated via solvent evaporation under vacuum over three days (100 mbar, 40 °C). Then, 100 μL were deposited over a nickel grid coated with formvar resin. Samples were examined with the electron microscope EM-1011 (JEOL, Tokyo, Japan) operating at 80 kV. Micrographs were taken with a Orius 1200A digital camera (Gatan, Pleasanton, CA, USA) using the DigitalMicrograph software package (Gatan, Pleasanton, CA, USA).

### 2.4. Cell Cultures and Transfection Experiments

The C2C12 mouse myoblast cell line (ATCC CRL-1772), was maintained in Dulbecco’s Modified Eagle’s Medium (DMEM) containing 4500 mg/mL glucose, 4 mM of L-glutamine, and 1 mM sodium butyrate (Sigma Aldrich, St. Louis, MO, USA) supplemented with 10% of heat-inactivated FBS (Gibco™—Thermo Fisher Scientific, Waltham, MA, USA), 50 I.U./mL penicillin–50 g/mL streptomycin (Gibco™—Thermo Fisher Scientific, Waltham, MA, USA), and 1 × MEM containing nonessential amino acids (Gibco™—Thermo Fisher Scientific, Waltham, MA, USA), and 1 × MEM containing nonessential amino acids (Gibco™—Thermo Fisher Scientific, Waltham, MA, USA) in a humidified incubator containing a 37 °C atmospheric O_2_/5% CO_2_ environment. For cell culture experiments, the cells were seeded uniformly onto sterilized coverslips in 12-well culture plates at 2 × 10^4^ cells per cm^2^. Cells were incubated 24 h until they reached 70% confluency. For nanocomplex transfections, C2C12 cells were washed twice with DPBS and kept 15 min in Opti-MEM (Invitrogen, Waltham, MA, USA) supplemented with 100 μg/mL of yeast t-RNA. C2C12 cells were exposed to PEI-cit/ASO/Ap nanocomplexes diluted in Opti-MEM or to 100 pmol of Cy3-conjugated aptamer for 3 h. Transfected cells were washed with DPBS to remove the excess of non-transfected nanocomplexes or free Cy3-conjugated aptamer.

For the colocalization assays, cells were fixed in 3.7% freshly prepared paraformaldehyde in PBS for 15 min under mild agitation. After several washes, DPBS samples were mounted onto slides with ProLong Anti-Fading Medium (Invitrogen, Waltham, MA, USA) formulated with DAPI (4′,6-diamidino-2-phenylindole) for DNA staining and examined with a Nikon A1R confocal scanning laser microscope. The transfection efficiency and colocalization coefficients were analyzed by the fluorescence detection of the Cy3 dye attached to the 5′ chain of the A01B aptamer and the FITC fluorophore linked to the PEI molecules.

For the cytotoxicity assays following PEI treatment, we analyzed the induction of apoptosis in C2C12 cultures exposed to an increasing amount of PEI (0–100 µg/mL) that were prepared as described above. DNA fragmentation was detected with a transferase-mediated dUTP nick end-labeling (TUNEL) method using a fluorescent staining kit according to the manufacturer’s instructions (Roche Diagnostics, Basel, Switzerland).

For GapmeR transfection experiments, a FAM-conjugated antisense LNA GapmeR negative control A (Ref. 339516) was purchased from Exiqon (Qiagen, Hilden, Germany). Lipofection was performed using Dhamafect-1 (Dharmacon™, Lafayette, CO, USA) following instructions from the manufacturer. The C2C12 cells were seeded at 12,500 cells/cm^2^, and two hours prior to transfection, the culture media was replaced with Opti-MEM I media without serum supplementation. GapmeR was also mixed with Opti-MEM I, and after 5 min of incubation at room temperature, the GapmeR solution was added dropwise on top of the Dharmafect solution and incubated for 20 min at RT. The mix was then added to the cells dropwise. Twenty-four hours after transfection, one volume of DMEM with 10% FBS and 1% penicillin–streptomycin was added to the wells and incubated at 37 °C for another 24 h. Forty-eight hours after transfection, cells were processed as described above for confocal analysis.

### 2.5. Immunofluorescence and Confocal Microscopy

Cells were fixed in 3.7% paraformaldehyde for 15 min and then permeabilized with 0.5% Triton X-100 in PBS for 15 min at room temperature. To identify cytoskeletal actin filaments, C2C12 samples were incubated with FITC-conjugated phalloidin (Sigma P5282) for 30 min. After several washes with PBS, the coverslips were mounted with ProLong with DAPI. The transfection efficiency of the internalized nanocomplexes was analyzed using confocal microscopy to analyze the Cy3 fluorescence intensity of the A01B signal within each FITC-phalloidin-stained cell. Confocal images were taken on a LSM510 (Zeiss, Jena, Germany) laser scanning microscope using a 63× oil (1.4 NA) objective and were processed using Adobe Photoshop CC 2021 software (v. 22.3.0. Adobe Systems, San José, CA, USA).

### 2.6. Transmission Electron Microscopy

C2C12 cells were cultured in 10-cm-diameter tissue culture plates and were allowed to grow until they reached 90% confluency. Then, the cells were transfected with PEI-cit/ASO/Ap nanocomplexes and synthetized by the CA technique following the steps aforementioned. After three hours, transfected cells were fixed for 45 min in 3% glutaraldehyde diluted in 0.12 M phosphate buffer (pH 7.4) containing 0.5% CaCl_2_. Cells were scraped off culture tissue plates and centrifuged for 10 min at 7000 rpm and subsequently 10 min at 12,000 rpm. Afterwards, cellular pellets were rinsed in 0.12 M phosphate buffer, post-fixed in 2% osmium tetroxide for 3 h, dehydrated in acetone, and embedded in araldite (Durcupan, Fluka, Buchs, Switzerland). Ultrathin sections were examined with a JEOL EM-1011 (Tokyo, Japan) electron microscope. Micrographs were taken with a camera (Orius 1200A; Gatan, Pleasanton, CA, USA) using the DigitalMicrograph software package (Gatan, Pleasanton, CA, USA). Electron micrographs were processed using Adobe Photoshop CC 2021 (v. 22.3.0. Adobe Systems, San José, CA, USA).

### 2.7. Quantification and Statistical Analysis

The colocalization ratio between the FITC nanocomplexes and Cy3-A01B aptamer and the mean area of the colocalizing nanoparticle micron-sized cytoplasmic domains (MSCD) per cell were quantified from at least 3 confocal micrographs of the transfection experiments. Using the image processing software Fiji ImageJ (U.S. National Institute of Health, Stapleton, NY, USA), the Mander’s colocalization coefficients and the pixel intensity histogram were obtained. Mander’s colocalization coefficient varies between 0 and 1 (a value of 1 means a perfect colocalization) and is defined as the colocalization degree of the pixels in a cooccurrence, i.e., the correlation between the green pixels that overlap with the ones in the red channel. The evaluation of the internalization efficiency of the different nanocomplex formulations (NP, ES, and CA) was analyzed studying: (i) the mean fluorescence intensity (MFI) of the MSCD per cell, following the procedure previously reported/procedures described in the literature [26], and (ii) the mean number of MSCD per cell. In both quantifications, the total number of quantified cells was counted from 3 micrographs for each synthesis technique and from 2 micrographs of aptamer-positive controls. All transfection experiments were performed/run in duplicate. Characterization data of the nanocomplexes synthetized by the three studied techniques and the cell internalization quantifications were expressed as the mean ± standard deviation (SD), and the statistical differences between multiple groups were assessed by one-way analysis of variance (ANOVA), followed by Tukey’s HSD (*n* ≥ 2). The statistical significance level was defined at * *p* < 0.05.

## 3. Results and Discussion

### 3.1. Comparison of PEI-Cit/ASO/Ap Nanocomplexes’ Synthesis Methodologies

**Figure 1:**
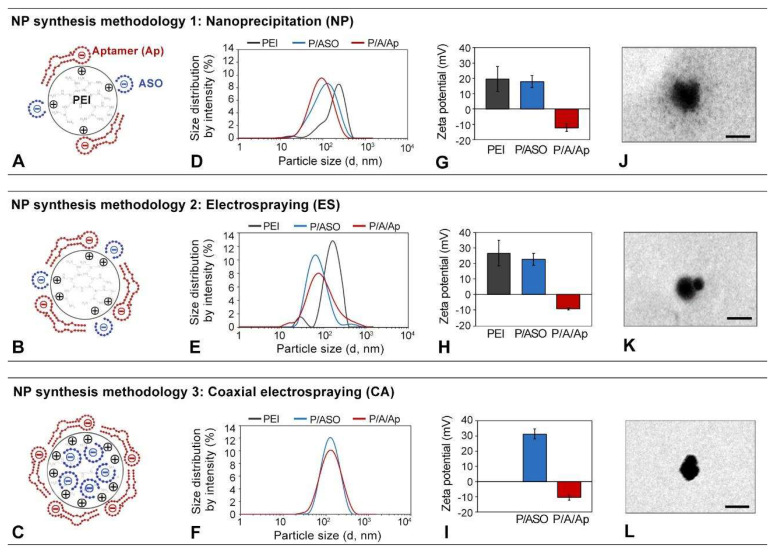
(**A**–**C**) Schematic depiction of the electrostatic structure of the PEI-cit/ASO/Ap nanocomplex attained with the three synthesis methodologies: nanoprecipitation (NP, (**A**)), electrospraying (ES, (**B**)), and coaxial electrospraying (CA, (**C**)). (**D**–**F**) Particle size distribution of the PEI-cit nanocomplexes at different levels of functionalization (PEI-cit, PEI-cit/ASO, and PEI-cit/ASO/Ap) synthesized by the three methodologies: NP (**D**), ES (**E**), and CA (**F**). (**G**–**I**) Z-potential of the surface of the PEI-cit nanocomplexes at different levels of functionalization (PEI-cit, PEI-cit/ASO, and PEI-cit/ASO/Ap) synthesized by NP (**G**), ES (**H**), and CA (**I**). Note the decrement of the Z-potential due to the addition of the electronegative oligonucleotides (ASO, Ap) to the positively charged nanocore. (**J**–**L**) TEM images of PEI-cit/ASO nanocomplex morphology by: NP (**J**), ES (**K**), and CA (**L**). Scale bars: 180 nm.

Figure 1A–C contains schemes representing PEI-cit-based nanocomplexes synthesized by either NP, ES, or CA, respectively. The electrostatic charge distribution, according to the final spatial configuration of the positively charged PEI polymer with the negatively charged ASO and aptamer molecules, is also depicted (Figure 1A–C). The particle size distributions and Z-potential mean values of the nanocomplexes were measured and statistically analyzed (Figure 1D–I). Finally, representative TEM images of the isolated PEI-cit/ASO nanocomplex are also shown (Figure 1J–L). Below, we analyzed our results of the nanocomplex synthesis methodology (NP, ES, or CA) and molecular composition: (i) PEI-citrate nanocomplexes (PEI-cit), (ii) PEI-cit carrying ASOs (PEI-cit/ASO), and (iii) PEI-cit/ASO functionalized with cell-specific RNA aptamers (PEI-cit/ASO/Ap).

First, PEI-cit nanocomplexes (black line) were synthesized by NP (Figure 1D) or ES (Figure 1E). These NP nanocomplexes were relatively polydispersed, with a broad particle size distribution (Figure 1D). The mean particle size and PDI (polydispersion index) of the NP nanocomplex dispersion were 359 ± 57 nm and 0.51 ± 0.08, respectively, which decreased to 162 ± 24 nm and 0.35 ± 0.08 for the ES nanocomplexes. These large PDI values of both samples represent a broad and polydisperse particle size distribution. The Z-potential of PEI-cit synthetized by ES was more positive than that synthetized by NP (+27 ± 8 mV vs. +20 ± 1 mV) (Figure 1G,H). The difference between both techniques could be attributed to the additional superficial charge offered by the positive electric field applied in the ES process [22,27]. This additional charge of the ES nanocomplexes may also favor its stability in dispersion and a lower aggregation against those produced by NP.

Secondly, an ASO molecule was incorporated to generate PEI-cit/ASO nanocomplexes by (i) external functionalization for the NP and ES techniques and (ii) encapsulation for CA. The integration of ASO molecules into the PEI-cit nanocomplexes, synthetized by either NP or ES, importantly reduced the mean particle sizes to 94 ± 4 nm and 90 ± 35 nm, respectively (Figure 1D,E; blue lines). This effect is probably attributed to a shorter reaction time of 5 min instead of 20 min for the PEI-cit nanocomplexes between the PEI and sodium citrate prior to the ASO addition, thus controlling the crosslinking of the polymer and, therefore, the particle size of the nanocomplexes [17]. PDI values of the PEI-cit/ASO nanocomplexes were also reduced to 0.34 ± 0.07 and 0.26 + 0.09 for NP and ES, respectively, when compared to PEI-cit. The external functionalization of PEI-cit with negatively charged ASO molecules subtly decreased the surface Z-potential: 18 ± 4 for NP and 23 ± 4 mV for ES (blue bars in Figure 1G,H) when compared to PEI-cit. Similar reductions in the Z-potential of similar PEI nanocomplexes were also reported after their external functionalization with RNA or oligonucleotide molecules [28,29,30]. Regarding CA-synthesized PEI-cit/ASO nanocomplexes, they presented a mean particle size of 116 ± 5 nm, a PDI of 0.17 ± 0.01 (Figure 1F), and a Z-potential of +31 ± 3 mV (Figure 1I). Noteworthy, the monomodal and stable dispersion of PEI-cit/ASO nanocomplexes, when synthetized by CA, suggests that the negatively charged ASO encapsulation produces the stabilization of the positively charged PEI and could avoid their agglomeration [31]. Furthermore, the highly positive Z-potential observed for the PEI-cit/ASO nanocomplexes by CA compared to NP and ES might indicate that encapsulated ASO could also reduce the crosslinking of PEI by sodium citrate. This hypothesis seems to also be supported by the absence in CA nanocomplexes of the grayish “cloud” that surrounds the PEI-cit/ASO nanocomplexes by NP and ES in the TEM images (Figure 1J–L) that can be attributed to the citrate crosslinking networks. The TEM images show spherical PEI-cit/ASO nanostructures with particle sizes in agreement with the DLS results. Overall, the mean particle sizes obtained by the three synthesis techniques agreed with other studies of PEI/RNA formulations (between 90 and 145 nm). [28,32] and within favorable sizes for cell transfection (20–200 nm) [11].

Lastly, a final functionalization of the PEI-cit/ASO nanocomplexes with RNA aptamer molecules (PEI-cit/ASO/Ap) did not significantly change the mean particle size and PDI (red lines in Figure 1D–F). However, the Z-potential values (Figure 1G–I) dropped sharply to approximately −10 mV in all cases, confirming the successful external functionalization of the nanocomplexes with the aptamer. Some authors showed the same sudden shift in the surface charges of the PEI-cit nanocomplexes, e.g., from 34.6 mV to −30.0 mV, when a siRNA and an aptamer were assembled on the PEI-cit nanocore [31]. The Z-potential drop from the PEI-cit/ASO to the PEI/cit/ASO/Ap nanocomplexes increased for the applied synthesis methodologies in the following order: NP (from 18 ± 4 to −12 ± 2 mV) < ES (from +23 ± 4 to −9 ± 1 mV) < CA (from +31 ± 3 to −10 ± 2 mV). This might indicate that CA provides the highest surface functionalization with the aptamer, given that, in NP and ES, ASO molecules compete with aptamer molecules for positively charged surface spots. Meanwhile, in CA, ASO cargo is encapsulated within the PEI-cit nanocomplexes; therefore, these nanocomplexes present a maximum positively charged surface area for the external electrostatic binding of the aptamer.

Interestingly, after 30 days of storage, the PEI-cit/ASO nanocomplex dispersions presented stable and monomodal (PDI values around 0.24 ± 0.06) particle distribution curves (Figure S2A–C). The nanocomplex agglomeration is undesirable for receptor-mediated transfection, as big, foreign bodies are prone to macrophage phagocytosis [33]. Hence, the excellent stability of the present PEI-cit/ASO nanocomplex dispersion confirms its ability to be stored and/or transported for long periods of time, preserving its functionality independently of the synthesis method employed.

### 3.2. In Vitro Transfection of PEI-Cit/ASO/Ap Nanocomplexes on Muscle Cells

First, we evaluated whether the cellular exposure to PEI caused cytotoxicity in the murine muscle model used in this work (C2C12 cell line). The TUNEL assay showed that less than 1% of C2C12 cells exposed for 3 h to increasing doses of PEI (0.1, 0.5, 1, 10, and 100 µg/mL) underwent apoptosis (TUNEL-positive) (Figure S1C). Next, to evaluate the molecular integrity of PEI-cit/ASO/Ap nanocomplexes during cellular transfection, a PEI polymer conjugated with fluorescein isothiocyanate (FITC) molecules was used to synthesize double-fluorescent FITC-PEI-cit/ASO/Ap-Cy3 nanocomplexes. FITC presents an emission wavelength of 512 nm that can be clearly distinguished from the Cy3 fluorochrome-labeled aptamer (570 nm). The stable covalent bonding of FITC with PEI amine groups also contributed to the selection of this fluorochrome [34,35]. Once PEI and FITC were conjugated, the efficiency of the conjugation was estimated at 90%, as measured by UV–Vis absorbance.

The methodology selected to synthesize FITC-PEI-cit/ASO/Ap-Cy3 nanocomplexes was CA, since it ensures ASO encapsulation and nanoparticle formation in a single procedure. The nanocomplexes were further functionalized with the Cy3-conjugated muscle-specific aptamer, as described for nonfluorescent PEI-cit/ASO/Ap nanocomplexes. DLS measurements of the FITC-PEI-cit/ASO/Ap-Cy3 nanocomplexes confirmed comparable mean particle size (151 ± 5 nm) and PDI (0.194 ± 0.015) values as nonfluorescent PEI nanocomplexes, and they were also stable during a storage period of at least 14 days (Figure S2D–F).

**Figure 2:**
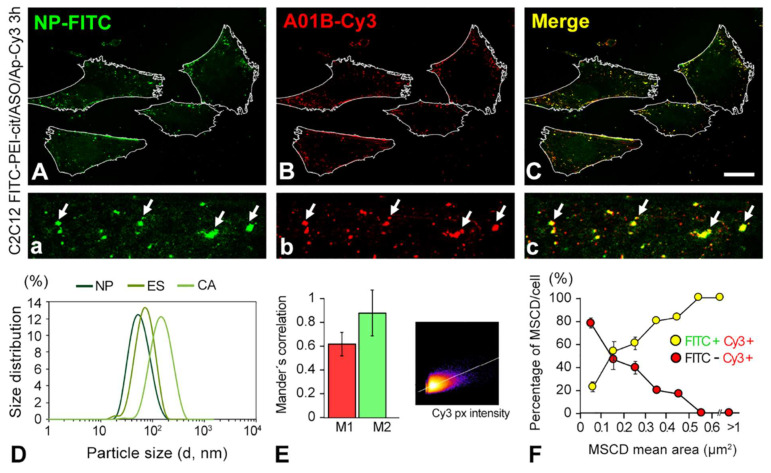
(**A**–**C**) Confocal microscopy images of C2C12 cells transfected with the FITC-PEI-cit/ASO/Ap-Cy3 nanocomplexes. Images show the colocalization from the PEI green (FITC) signal (**A**) and the aptamer (Ap) red signal (Cy3) (**B**), as well as (**C**) the overlap of both channels within the cells. Note that in the higher magnification details (**a**–**c**), only the micron-sized structures of both channels, denoted by arrows, colocalized (yellow). (**D**) Particle size distribution curves of the fluorescent nanocomplexes (FITC-conjugated PEI) synthetized by the three studied synthesis methodologies (NP, ES and CA). (**E**) Colocalization analysis of the endocytic nanocomplexes transfected in C2C12 cells by Mander’s colocalization coefficients (M1 and M2) showing the amount of fluorescence of the pixels in cooccurrence in both color channels (green channel, FITC-PEI; red channel, Ap-Cy3), and a representative colocalization intensity histogram of the confocal microscopy images. (**F**) Percentage of the nanoparticle-loaded vesicles (MSCD) per cell vs. their mean area. Note that the percentage of the colocalizing MSCD per cell increased as the MSCD size became larger. Scale bar: 15 µm (**A**–**C**).

To evaluate the stability of the nanocomplex after cellular uptake, we transfected C2C12 cells with double-fluorescent-labeled FITC-PEI-cit/ASO/Ap-Cy3 nanocomplexes. Using high-resolution confocal images, we analyzed the degree of colocalization of the polymer (FITC) and aptamer (Cy3) intracellular signals. Figure 2A–C clearly illustrate the strong colocalization of the green signal (PEI) with the red signal (aptamer). The quantitative estimation of the colocalization ratio between FITC-PEI-cit and Ap-Cy3 was determined by analyzing the overlapped pixels of the fluorescent micron-sized structures on confocal images recorded at the highest magnification (Figure 2A–C) and calculating the Mander’s colocalization coefficient (Figure 2E). This coefficient quantifies the cooccurrence between two fluorescent molecules (e.g., green and red channel signals) present in one or more regions of interest (ROIs), i.e., the number of cooccurring pixels as a fraction of the total number of pixels. Mander’s coefficient is almost independent of the signal intensities but is very sensitive to pixel colocalization in both channels [36]. This coefficient is commonly expressed by two complementary coefficients, M1 and M2, which represent, separately for each fluorochrome, the pixel fraction of the total fluorescence that colocalizes. In this study, M1 is the number of red pixels that overlaps with the green channel, and M2 is the number of green pixels that overlaps with the red channel [37,38]. The M2 coefficient revealed a ratio of 0.89 ± 0.19, higher than the M1 coefficient (0.62 ± 0.10), revealing that almost all the green pixels colocalized with the red pixels. This was also confirmed with the pixel intensity scatterplot (Figure 2E inset) that followed a congruent fitting to the linear increase attributed to the overlap of red and green pixel intensities. Moreover, it is noteworthy that most of the colocalization foci (yellow signal) belonged to micron-sized cytoplasmic domains (MSCD) with high polymer (green signal) and aptamer (red signal) concentrations (Figure 2A–C, denoted by white arrows). Meanwhile, submicron fluorescent red spots, which did not colocalize with the green polymeric fluorescent signal, were also visualized.

Furthermore, we used 5′-FITC-conjugated ASOs to synthetize PEI-cit/FITC-ASO/Ap-Cy3 double-fluorescent-labeled nanocomplexes. Cells were exposed to these nanoparticles for 3 h and analyzed by confocal microscopy. Our analysis revealed the presence of both green and red signals (ASO and Aptamer) within the cells after 3 h of transfection treatment. This result further confirms that the synthesis procedure incorporates the ASOs to the multimolecular structure of the nanocomplexes and that these are capable of incorporating the ASOs by endocytosis in cultured C2C12 cells (Figure S3A,B).

Taken together, these results confirm the intracellular presence of integral FITC-PEI-cit/ASO/Ap nanocomplexes and indicate that Ap functionalization is preserved after nanocomplex uptake by C2C12 muscle cells. Figure 2F describes the percentage per cell of MSCD (positive to both red and green signal channels) and of Aptamer-enriched foci (only positive to the red signal channel) distributed by the size average. It was found that large Cy3-positive areas (>0.5 µm^2^) mainly corresponded to MSCD (>90%). Conversely, smaller Cy3-positive areas (<0.5 µm^2^) might only contain free aptamer (the absence of the green signal). These findings allowed us to set a minimum threshold, based on the size of Cy3-positive areas, to quantify the transfection efficiency of integral PEI-cit/ASO/Ap-Cy3 nanocomplexes using Cy3-based densitometric image analysis.

### 3.3. Ultrastructure of the Cellular Mechanism of Nanocomplex Incorporation into Muscle Cells

**Figure 3:**
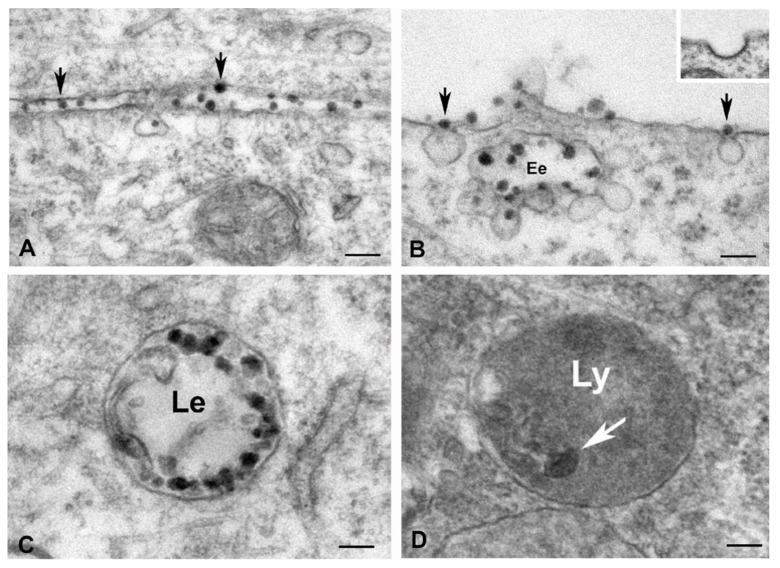
(**A**–**D**) Electron microscopy illustrating the endocytic pathway of the PEI-cit/ASO/Ap nanocomplexes in cultured myoblasts. (**A**) Interactions of nanocomplexes (electron dense particles) with the plasma membrane (arrows). (**B**) Incorporation of nanocomplexes in non-coated endocytic vesicles (arrows). Note the fusion of internalized vesicles containing nanocomplexes with an early endosome (Ee). Inset: Detail of a clathrin coated vesicle. (**C**) Late endosome (Le) loaded with numerous electron dense particles of nanocomplexes. (**D**) Electron dense body of lysosomal nature containing residual nanocomplexes. Scale bars: 100 nm (**A**–**C**) and 50 nm (**D**).

Regarding the transfection mechanism of the nanocomplexes, previous studies reported that the cellular uptake of nanoparticles was performed via cell endocytosis [8,39]. Our transmission electron microscopy analysis clearly demonstrated that PEI-cit/ASO/Ap nanocomplexes are internalized into cells following the endocytic and endosomal trafficking pathways [40,41] (Figure 3A–D). This is an important point given that not all cells have the same internalization machinery. Therefore, understanding the cellular uptake mechanism is essential for producing nanocomplexes that are engineered to target specific cellular pathways [42].

PEI-cit/ASO/Ap nanocomplexes were clearly visualized as electron-dense particles, ranging in diameter from 40 to 60 nm. Importantly, all sequential steps of the endocytic pathway were clearly recognized by ultrastructural analysis (Figure 3A–D). First, PEI-cit/ASO/Ap nanocomplexes directly interact with the plasma membrane (Figure 3A,B), overcoming the anionic barrier of the glycocalyx, a molecular network of carbohydrate residues from plasma membrane glycoproteins and glycolipids [5]. Second, there was the formation of nanocomplexes containing pits that invaginate to form noncoated vesicles, which pinches off the plasma membrane for releasing into the cytoplasm (Figure 3A,B). Interestingly, the absence of a coat on the cytosolic surface of these vesicles (Figure 3B, inset) suggests that PEI-cit/ASO/Ap nanocomplexes are internalized by clathrin-independent endocytosis [43]. Third, there was the fusion of endocytic vesicles carrying PEI-cit/ASO/Ap nanocomplexes to early endosomes beneath the plasma membrane (Figure 3B). Fourth, there was the maturation of early endosomes in late endosomes that were loaded with numerous electron-dense particles of PEI-cit/ASO/Ap nanocomplexes (Figure 3C). Some residual electron dense particles were observed in cytoplasmic bodies identified as secondary lysosomes (Figure 3D).

Once the PEI-cit/ASO/Ap nanocomplexes are internalized via endocytosis, (Figure 3B), ASOs must escape from early and late endosomes to reach RNA targets in the cytosol or nucleus. Previous studies have identified several factors, including proteins ANXA2 (Annexin A2), AP2M1 (Adaptor-related protein complex 2 subunit mu 1) and the endosomal GTPase Rab5C, as well as the acidification of vesicle compartments, that facilitate endocytic trafficking and release of ASOs from endosomal compartments into the cytoplasm [8,44,45]. Moreover, other data have shown that lysophosphatidic acid is required for release phosphorothioate-modified ASOs from the late endosome into the cytosol [46].

The cellular uptake of nanocomplexes by macropinocytosis—the mechanism of enclosure and uptake by endocytosis of large volumes of extracellular medium [42]—was not observed. The ultrastructural organization and distribution of the endocytic network of PEI-cit/ASO/Ap-loaded vesicles in discrete regions of the cell cortex or cytoplasm interior must correspond to the MSCD with high polymer and aptamer concentrations, as previously described (Figure 2F).

### 3.4. Methodological Comparison of Functionalized Nanocomplexes to Induce Cellular Uptake by Endocytosis

**Figure 4:**
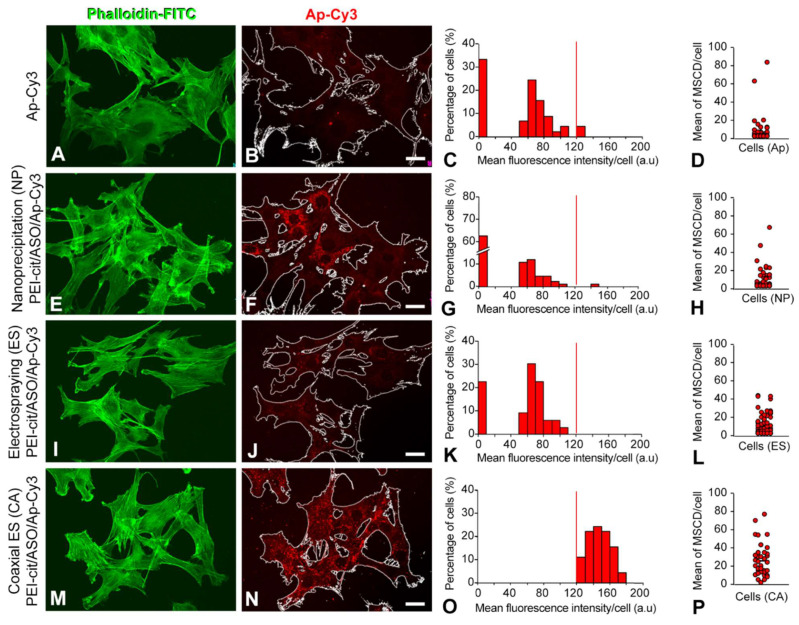
(**A**,**E**,**I**,**M**) Confocal images showing the actin filaments (FITC-conjugated phalloidin) of the cytoskeleton from C2C12 cells. (**B**,**F**,**J**,**N**) Analogous confocal images showing the same C2C12 cells outlined transfected with either free Cy3-conjugated aptamer (**B**) or with the PEI-cit/ASO/Ap-Cy3 nanocomplexes synthetized by nanoprecipitation ((**E**), NP), electrospraying ((**I**), ES), and coaxial electrospraying ((**N**), ES). (**C**,**G**,**K**,**O**) Mean fluorescence intensity histogram of the Cy3 aptamer signal only from nanocomplexes located at MSCD. Vertical red lines correspond to the threshold limit that discriminates the MFI corresponding to MSCD of areas above and below 0.5 µm^2^. (**D**,**H**,**L**,**P**) Dispersion diagram of the mean number of MSCD of mean area ≥ 0.5 µm^2^ per cell. Note that most cells transfected with free aptamer (**D**), do not exhibit MSCD. Conversely, all cells transfected with CA nanocomplexes enclosed at least 1 MSCD in their cytoplasm. Scale bar: 20 µm.

The efficiency of C2C12 transfection by the PEI-cit/ASO/Ap nanocomplexes synthesized by the three different methodologies (NP, ES, and CA) is compared in Figure 4A–N. Foremost, to test whether treatment with nanocomplexes induced morphological changes in C2C12 cells, cultured cells were stained with phalloidin-FITC, a cytochemical marker of F-actin filaments. After 3 h of treatment, confocal analysis (Figure 4E,I,M for NP, ES, and CA, respectively) revealed that C2C12 cells preserved the same polygonal shape that was observed in the positive control (treatment with free Ap-Cy3, Figure 4A). Moreover, the administered dose of nanocomplexes did not appear to cause any cytotoxic effect on the cell cultures after 3 h of treatment. Mean fluorescence intensity (MFI) measurements of the Ap-Cy3 positive foci with mean area values ≥0.5 µm^2^ were carried out using ImageJ software following the procedure illustrated in Figure S3C–J. The sole transfection of the Ap-Cy3 in C2C12 cells allowed us to quantify the MFI background for the red channel since the appearance of Cy3-positive foci was very low (Figure 4B–D).

Regarding PEI-cit/ASO/Ap transfection, significant differences were observed depending on the nanocomplex synthesis methodology employed. The majority of C2C12 cells presented a low number of MSCDs when exposed to NP PEI-cit/ASO/Ap nanocomplexes. However, a small percentage (<5%) of the cells analyzed showed a mean fluorescence signal above the background corresponding to numerous MSCDs per cell (Figure 4F–H). This was indicative of a non-homogeneous cell transfection of NP PEI-cit/ASO/Ap nanocomplexes. Meanwhile, the ES technique (Figure 4J–L) presented homogeneous nanocomplex transfection from cell to cell; however, the mean number of MSCDs per cell was very reduced. Remarkably, the PEI-cit/ASO/Ap nanocomplexes synthesized with CA technology (Figure 4N–P) significantly exhibited an important transfection capacity in terms of the number of MSCDs per cell, the mean fluorescence intensity of MSCDs per cell, and the cell-to-cell MSCD homogeneity, as most of the cells presented a similar mean number of MSCD (≥20) per cell.

### 3.5. Functionalized Nanocomplexes Synthesized by Coaxial Electrospraying Are Able to Efficiently Deliver RNA-Based Therapies into Muscle Cells

**Figure 5:**
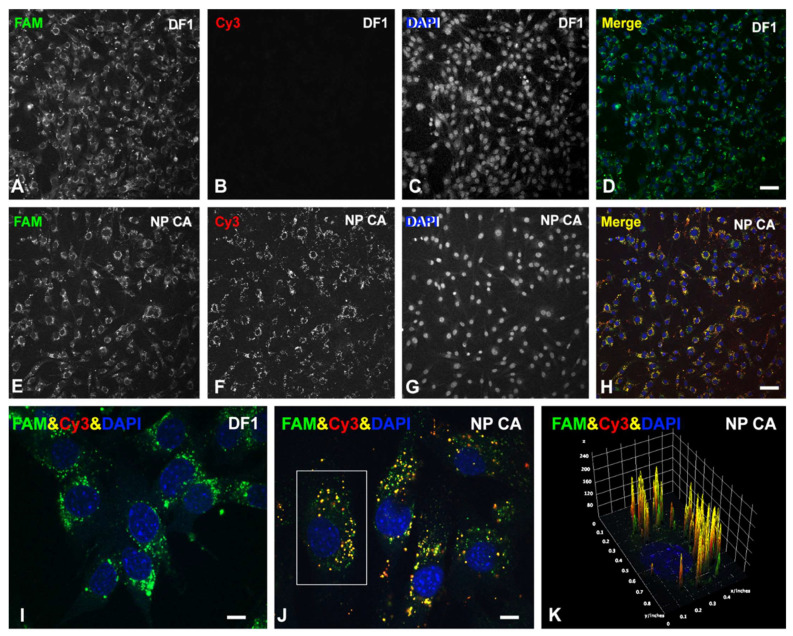
Confocal images of C2C12 cells showing the cellular uptake of a FAM-conjugated non-targeting GapmeR (FAM, green) achieved using either Dharmafect 1 (DF1) as RNA delivery liposomal-based methodology (DF1) (**A**–**D**) or by a muscle receptor-mediated mechanism with CA synthesized nanocomplex functionalized with the Cy3-conjugated specific A01B aptamer (Cy3, red) (**E**–**H**). Cells were fixed and counter stained with DAPI (blue) to visualize cell nuclei. (**I**,**J**) Higher magnification (100×) confocal images showing the cytoplasmic FAM-GapmeR accumulation, surrounding the cell nuclei, using both DF1 (**I**) and CA nanocomplexes (**J**). Note the strong colocalization of FAM-GapmeR with the Cy3-aptamer in yellow foci, corresponding to MSCD. (**K**) Fluorescence intensity analysis using 3D interactive surface plot from the Fiji software from the cell indicated in ((**J**), white box). The graphical representation allows a clear visualization of intracellular FAM-conjugated Gapmers (green) and Cy3-conjugated Aptamer (red). Each yellow peak represents Gapmer/Aptamer (FAM/Cy3) colocalization in MSCD. Scale bars: 100 µm (**A**–**H**) and 15 µm (**I**–**J**).

To confirm that the PEI-cit/ASO/Ap-Cy3 nanocomplexes synthesized by CA are a good methodology to deliver therapeutic ASO molecules, nanocomplexes were reformulated to substitute the ASO molecule by a non-targeting GapmeR conjugated to a FAM fluorophore for fluorescence tracing. The transfection ability of these PEI-cit/FAM-GapmeR/Ap nanocomplexes was compared with the conventional RNA delivery, liposomal-based methodology Dharmafect (DF1) as a positive control of efficient transfection reagent. Following a conventional DF1-based transfection protocol, C2C12 cells were exposed to the FAM-GapmeR (150 nM) over 3 days. Figure 5A–D shows the successful cellular uptake of FAM-GapmeR. As observed, a strong and cell-to-cell equivalent green fluorescent signal from the GapmeR (FAM) was present in nearly 100% of the cells from the image field. Moreover, C2C12 cells exposed to the CA PEI-cit/FAM-GapmeR/Ap nanocomplexes (Figure 5E–H) exhibited the green signal from FAM-GapmeR and the red signal from Ap-Cy3. Using both transfection methodologies, DF1 and CA, C2C12 was seen to uptake the GapmeR molecules with comparable transfection efficiencies (Figure 5A,E). Figure 5I,J show, with higher magnification detail, the cytoplasmic accumulation of FAM-GapmeR into MSCDs, surrounding the nuclei, transfected with both DF1 and CA nanocomplex methodologies. Particularly, the colocalization of the green and red channels (yellow signal) in Figure 5J,K confirms the sustained stability of CA nanocomplexes during the entire intracellular incorporation process via late/early endosomes.

Nowadays, several therapeutic ASOs are regulatorily approved for treating different types of genetic diseases by the Food and Drug Administration (FDA) [47], such as, for instance, Nusinersen (Spinraza), a completely modified 2′-MOE therapeutic RNA-like ASO approved in 2016 to treat all types of Spinal Muscle Atrophy (SMA) [48,49]. The current clinical treatment for SMA is the intrathecal administration of Nusinersen, which specifically binds to the *SMN2* pre-mRNA and modulates its splicing in order to increase functional SMN protein, the product of the genetic cause of SMA [50,51]. A limiting factor of this ASO-based gene therapy for the treatment of SMA is the lack of cell specificity in myofibers [52]. Nowadays, Nusinersen is only administered intrathecally, so only affected spinal motor neurons are being treated [53]. A systemic administration would require very high doses of the drug, which would lead to an unpredictable expression of SMN and potential adverse effects in different organs, in addition to the risk of toxicity. Therefore, a non-specific interaction of ASOs with non-target cells would imply a significant loss of therapeutic molecules destined for myofibers and, consequently, of therapeutic efficacy. Therefore, at least in the SMA field, it is urgent to develop new therapeutic strategies directed to specifically deliver RNA-therapeutics to muscle cells. Nanoparticles based on cationic polymers, such as those presented herein, could be a good alternative. To the extent of our knowledge, RNA-based therapies using targeted delivery with nanoparticles for SMA treatment have not been reported so far. Thus, coaxially electrospun PEI-cit/Nusinersen/A01B-aptamer nanocomplexes could facilitate the effective delivery of the drug into SMA-affected muscle tissues.

## 4. Conclusions

This work shows that PEI-citrate nanoparticles made by coaxial electrospray and decorated with an aptamer can be used for the target delivery of ASOs into cultured muscle cells with similar efficiency as the standard transfection methods. Thus, using this methodology, we obtained a multi-molecular nanocomplex with a high rate of cellular uptake by muscle C2C12 cells via the activation of the clathrin-independent endocytic pathway. Therefore, this result could constitute the basis of in vivo tissue-specific ASO delivery systems. Future experiments will be devoted to the evaluation of the drug release of such CA nanocomplexes and their use as carriers of the ASOs-based therapeutic Nusinersen to animal models of congenital myopathy SMA.

## Data Availability

The data are available from the corresponding author upon reasonable request.

## Supplementary Materials

The following supporting information can be downloaded at: https://www.mdpi.com/article/10.3390/biom12081012/s1, Figure S1: PEI-cit/ASO/Ap nanocomplexes synthesis diagrams and citotoxicity assay. Figure S2: Stability assays of the PEI-cit/ASO/Ap nanocomplexes over 7, 14 and 30 days. Figure S3: Incorporation of PEI-cit/FITC-ASO/Ap nanocomplexes in C2C12 cells. Image-based procedure used to quantify the mean fluorescence intensity (MFI) of micron sized cytoplasmic domains (MSCDs) from C2C12 cells exposed to PEI-cit/ASO/Ap-Cy3 nanocomplexes.
