# Supplementary material for: Coaxial Synthesis of PEI-Based Nanocarriers of Encapsulated RNA-Therapeutics to Specifically Target Muscle Cells
Source: Biomolecules. 2022 Jul 22;12(8):1012. doi: 10.3390/biom12081012 (PMC9332584; doi:10.3390/biom12081012)
Supplement: Supplementary file 1 [file biomolecules-12-01012-s001.zip › biomolecules-1789304-supplementary.pdf]

biomolecules

MDPI

Article

Coaxial Synthesis of PEI-Based Nanocarriers of Encapsulated RNA-Therapeutics to Specifically Target Muscle Cells

Raquel de la Hoz [1,2], Nazely Diban [1,2,*], María T. Berciano [2,3,4], Carlos San Emeterio [5], Ane Urtiaga [1,2], Miguel Lafarga [2,4,6], José C. Rodríguez-Rey [2,3] and Olga Tapia [5,*]

[1] Department of Chemical and Biomolecular Engineering, University of Cantabria, 39011 Santander, Spain; raquel.delahoz@unican.es (R.d.l.H.); ana.urtiaga@unican.es (A.U.)
[2] Health Research Institute Valdecilla (IDIVAL), 39011 Santander, Spain; berciant@unican.es (M.T.B.); lafargam@unican.es (M.L.); josecarlos.rodriguez@unican.es (J.C.R.-R.)
[3] Department of Molecular Biology, University of Cantabria, 39011 Santander, Spain.
[4] "Centro de Investigación Biomédica en Red Sobre Enfermedades Neurodegenerativas" (CIBERNED), 28029 Madrid, Spain.
[5] Research Group on Food, Nutritional Biochemistry and Health. Universidad Europea del Atlántico, 39011 Santander, Spain.
carlos.sanemeterio@alumnos.uneatlantico.es)
[6] Department of Anatomy and Cellular Biology, University of Cantabria, 39011 Santander, Spain.

* Correspondence: nazely.diban@unican.es (N.D.); olga.tapia@uneatlantico.es (O.T.)

Supplementary Figures

Supplementary Figure S1

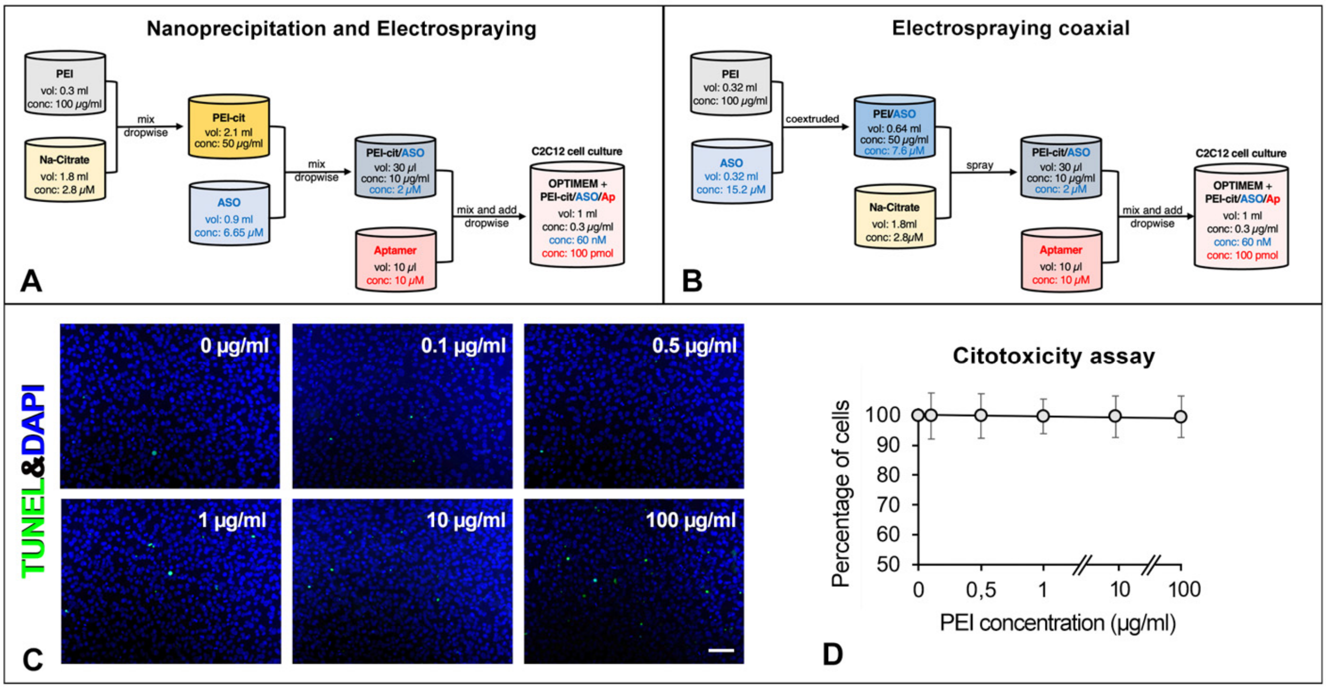

Figure S1. (A-B) Diagrams depicting the stepwise procedure for the PEI-cit/ASO/Ap nanocomplexes synthesis, by either NP or ES **(A)** and coaxial **(B)**. The final dilution step in OptiMEM for cellular uptake experiments is also indicated. Volumes used in each step as well as the concentration of PEI (**black**), ASO (**blue**) and Aptamer (**red**) is indicated for each solution. **(C)** Citotoxic assay based on TUNEL labeling, indicative of any kind of cell death. TUNEL labels 3′OH

DNA termini in DNA within whole and fragmented nuclei. TUNEL assay was performed after treatment of C2C12 cells with the amount of PEI indicated for 3 hours. Cells were counstained with DAPI to quantify the total amount of cells. TUNEL-positive cells were quantify and the mean percentage of TUNEL-negative cells per image field was calculated and plotted against PEI concentration **(D)**. Dots represent mean ± SD of at least 3 images. Scale bar: 150µm

Supplementary Figure S2

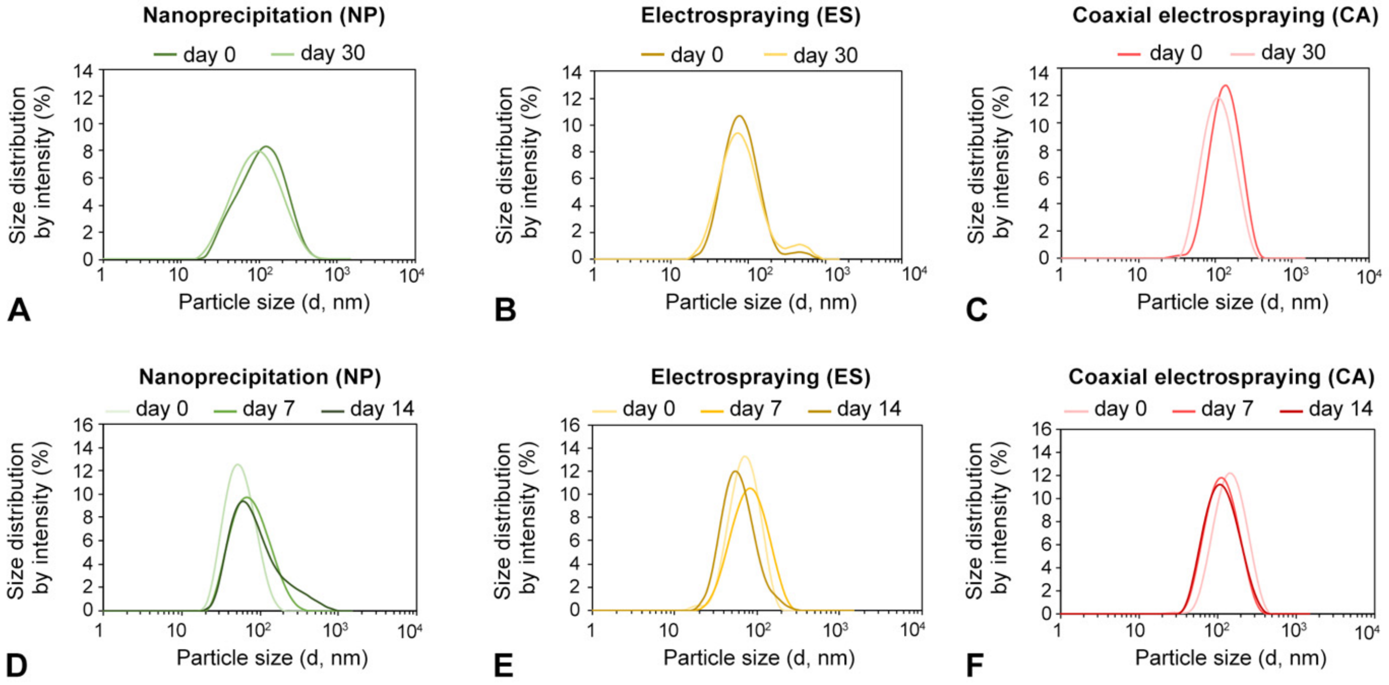

Figure S2. (A-C) Stability assays, over 30 days, showing the particle size distribution curves (by DLS) of the PEI-cit/ASO nanocomplexes with the three synthesis methodologies: nanoprecipitation (NP, **A**), electrospraying (ES, **B**) and coaxial electrospraying (CA, **C**). **(D-F)** Stability assays, over 7 and 14 days, showing the particle size distribution curves of the PEI-FITC-cit/ASO nanocomplexes with the three synthesis methodologies: NP **(D)**, ES **(E)** and CA **(F)**.

Supplementary Figure S3

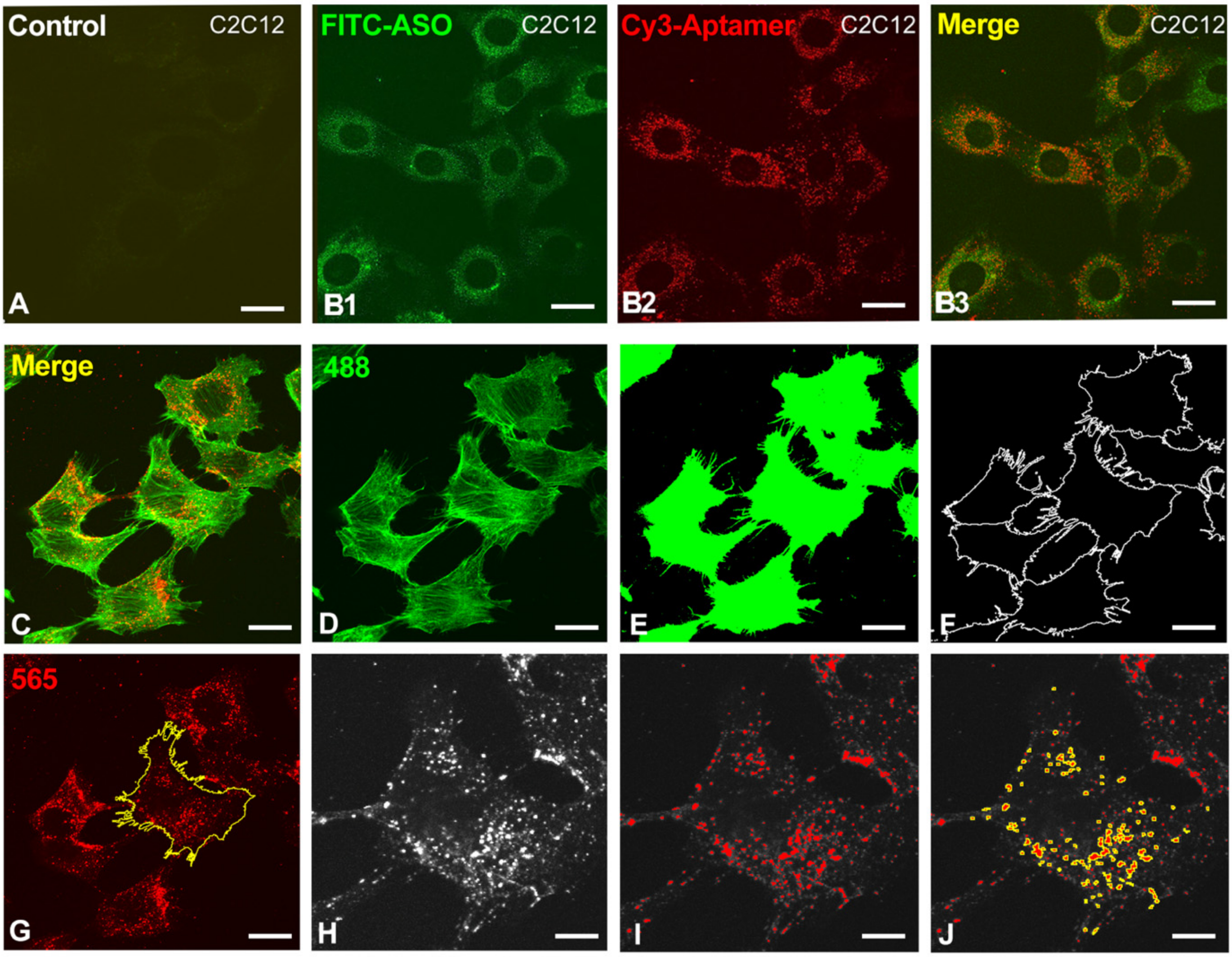

Figure S3. (A) Image of C2C12 mouse myoblasts not treated with PEI-cit/ASO/Ap nanoomplexes. **(B1, B2, B3)** Confocal imaging of C2C12 myoblasts illustrating the incorporation of nanocomplexes containing the FITC-conjugated ASOs (FITC, green) (**B1**) and the Cy3-conjugated Aptamer A01B (Cy3, red) **(B2). (E3)** Merge channel. **(C-J)** Confocal microscopy image-based procedure used using ImageJ software to quantify the mean fluorescence intensity (MFI) of micron sized cytoplasmic domains (MSCDs) from C2C12 cells exposed to CA-synthetized PEI-cit/ASO/Ap-Cy3 nanocomplexes. **(C)** Original RGB multi-stack confocal image (green and red channels merged). **(D)** F-actin filaments of C2C12 cells counterstained with FITC-Phalloidin (FITC-488nm, green channel). **(E)** Adjustment of brightness and contrast of green channel to its maximum values to clearly distinguish the cell body. **(F)** Green channel view showing the drawing of all the cell outlines (regions of interest, ROIs) loaded to the ROIs manager tool of ImageJ. **(G)** Aptamer-Cy3 signal (Cy3-565nm, red channel) of the transfected nanocomplexes inside the MSCDs and the selection of the previously loaded cell outlines in the ROIs manager. **(H)** Conversion of the red channel image into an 8-bit image. **(I)** Adjusting the red channel to uniquely select MSCD by using "Threshold" tool, loaded to the ROI manager tool of ImageJ. **(J)** MFI analysis of those MSCD with a mean area above 0.5 μm^2 (outlined in yellow) selected using the "Analyse particles" tool of ImageJ. Scale bar: 20μm (A-G) and 5μm (H-J).
